# Investigating causal links between gallstones, cholecystectomy, and 33 site-specific cancers: a Mendelian randomization post-meta-analysis study

**DOI:** 10.1186/s12885-024-12906-2

**Published:** 2024-09-27

**Authors:** Fei Teng, Youyin Tang, Zhangyu Lu, Kefei Chen, Zheyu Chen

**Affiliations:** 1 Division of Liver Surgery, Department of General Surgery, West China Hospital, Sichuan University, No. 37 GuoXue Alley, Chengdu, 610041 China; 2 Division of Vascular Surgery, Department of General Surgery, West China Hospital of Sichuan University, No. 37 GuoXue Alley, Chengdu, 610041 China; 3 West China School of Medicine, Sichuan University, No. 17 South Renming Road, Chengdu, 610094 China

**Keywords:** Gallstones, Cholecystectomy, Small intestine tumors, Nonmelanoma, Multivariate mendelian randomization

## Abstract

**Background and aim:**

The association between gallstones/cholecystectomy and cancer remains inconclusive in the current literature. This study aimed to explore the causal connections between gallstones/cholecystectomy and cancer risk by utilizing a bidirectional two-sample multivariable Mendelian randomization approach with Genome-Wide Association Studies data.

**Methods:**

Utilizing Genome-Wide Association Studies data from the UK Biobank and FinnGen, this research employed multivariable Mendelian randomization analyses to explore the impact of gallstones and cholecystectomy on the risk of 33 distinct cancer types. Instrumental variables for gallstones and cholecystectomy were carefully selected to ensure robust analyses, and sensitivity and heterogeneity tests were conducted to verify the findings’ validity.

**Results:**

Multivariable Mendelian randomization analysis, incorporating data from more than 450,000 individuals for gallstones and cholecystectomy, revealed nuanced associations with cancer risk. Cholecystectomy was associated with a significantly increased risk of nonmelanoma skin cancer (OR = 1.59, 95% CI: 1.21 to 2.10, *P* = 0.001), while gallstones were linked to a decreased risk of the same cancer type (OR = 0.63, 95% CI: 0.47 to 0.84, *P* = 0.002). Interestingly, the analysis also suggested that cholecystectomy may lower the risk of small intestine tumors (OR = 0.18, 95% CI: 0.043 to 0.71, *P* = 0.015), with gallstones showing an inverse relationship, indicating an increased risk (OR = 6.41, 95% CI: 1.48 to 27.80, *P* = 0.013).

**Conclusions:**

The multivariable Mendelian randomization analysis highlights the differential impact of gallstones and cholecystectomy on cancer risk, specifically for nonmelanoma skin cancer and small intestine tumors. These results underscore the importance of nuanced clinical management strategies and further research to understand the underlying mechanisms and potential clinical implications of gallstone disease and cholecystectomy on cancer risk.

**Supplementary Information:**

The online version contains supplementary material available at 10.1186/s12885-024-12906-2.

## Study Highlights

**What is known**

1. Gallstone disease is a prevalent disorder of the gastrointestinal system that contributes significantly to healthcare expenditures annually.

2. Previous studies have reported inconsistent findings regarding the association between gallstones/cholecystectomy and various extrabiliary malignancies.

**What is new here**

1. In this study, multivariate Mendelian analysis revealed intricate and nuanced causal relationships between gallstones/cholecystectomy and pan-cancers, particularly extrabiliary tumors.

2. The study demonstrated that gallstone diseases were associated with a reduced risk of nonmelanoma (OR = 0.63, 95% CI: 0.47 to 0.84, *P* = 0.002), while cholecystectomy increased the risk (OR = 1.59, 95% CI: 1.21 to 2.10, *P* = 0.001).

3.The findings indicated that gallstone disease increased the risk of small intestinal neoplasms (OR = 6.41, 95% CI: 1.48 to 27.80,*P* = 0.013), whereas cholecystectomy decreased the risk (OR = 0.18, 95% CI: 0.043 to 0.71, *P* = 0.015).

**Supplementary Information:**

The online version contains supplementary material available at 10.1186/s12885-024-12906-2.

## Introduction

Gallstones, also known as cholelithiasis, are prevalent digestive disorders in Western countries, affecting up to 20% of the population [1]. The incidence of gallstones increases progressively with age [2, 3]. Prolonged gallstone formation can lead to gallbladder cancer development, with curative resection possible in only about 25% of diagnosed cases. The majority of patients require systemic treatment [4–7]. In Western countries, cholesterol constitutes over 90% of gallstones, contrasting with black pigment stones which represent less than 10% [8]. Risk factors for cholesterol gallstones include obesity, diabetes mellitus, elevated body mass index, female sex, biliary stasis, and decreased levels of high-density lipoprotein cholesterol due to physical inactivity and a sedentary lifestyle [9–11]. Conversely, black pigment stones primarily arise from hemolytic disorders and liver cirrhosis conditions [12, 13]. Furthermore, a prospective study indicated associations between gallstones, cholecystectomy, late menarche onset, and self-reported stress. Symptomatic gallstones are typically treated with cholecystectomy, performed in about 20% of cases [3, 14, 15]. However, the actual resection rate may be higher, as many patients with asymptomatic gallstones also undergo surgery [16–18]. These findings underscore the systemic implications of gallstones and the potential physiological alterations induced by cholecystectomy, potentially correlating with tumor development in various anatomical regions [16–18].

Bile acids, the primary constituents of bile, play a pivotal role in lipid digestion and absorption within the intestines. The human body excretes only 5% of bile acids daily through the enterohepatic circulation [19]. Additionally, the activation of the farnesoid X receptor by bile acids in intestinal epithelial cells triggers the expression of fibroblast growth factor 15/19, which subsequently suppresses cholesterol 7 α-hydroxylase expression in liver cells upon the entry of portal vein blood into hepatic sinusoids [20]. This intricate negative feedback mechanism ultimately governs the regulation of bile acid synthesis. In patients diagnosed with gallstones who have undergone surgical cholecystectomy, there is an increase in the total bile acid content within the intestines [19]. However, excessive accumulation of bile acids can stimulate the synthesis of secondary bile acids, such as deoxycholic acid (DCA) and lithocholic acid, leading to subsequent oxidative metabolic damage [21]. High levels of DCA can also modulate the composition of the gut microbiota, suppress FXR activity in small intestinal epithelial cells, and sustainably activate the Wnt/β-catenin signaling pathway associated with carcinogenesis, thereby promoting the development of colorectal cancer [22, 23].

Despite the potential for increased burden on intestinal liver circulation and oxidative metabolic damage resulting from gallstones or cholecystectomy, the link to subsequent malignant tumor development remains uncertain. Previous studies have established a correlation between the presence of gallstones or prior cholecystectomy and cancer, excluding gallbladder cancer and malignancies in the biliary tract [24, 25]. A large-scale observational study indicated an increased risk of kidney cancer among individuals who had undergone cholecystectomy within six months of surgery and before reaching 40 years of age [26]. Other investigations have also noted elevated risks of gastric cancer, right-sided colon cancer, and other malignancies following gallstone disease or cholecystectomy [27, 28]. However, not all studies demonstrate a correlation between cholecystectomy and subsequent malignancy incidence [29, 30]. The inconclusive findings from these studies have led to confusion regarding the association between gallstones/cholecystectomy and malignancy. It is important to note that confirming an increased risk of specific malignant tumors associated with gallstones or cholecystectomy would necessitate multiple large-scale, long-term prospective randomized controlled trials, which are both time-consuming and costly. Nonetheless, the use of Mendelian randomization analysis methods could potentially explore the causal association between gallstones/cholecystectomy and subsequent malignancy development.

Mendelian randomization (MR) is an epidemiological research method that leverages appropriate single nucleotide polymorphisms (SNPs) as instrumental variables to provide robust causal evidence regarding the relationship between exposure and outcome, based on Mendel’s law of independent assortment [31]. The instrumental variable (IV) approach allows MR to emulate randomized controlled trials by randomly assigning SNPs during mitosis, effectively mitigating confounding factors [32]. Furthermore, multivariable Mendelian randomization analysis was utilized to investigate the independent associations of gallstone disease and cholecystectomy with cancer development, as well as to explore potential interactions between these two factors [33].

Consequently, we conducted a bidirectional two-sample multivariable Mendelian randomization (MVMR) analysis to investigate the causal relationship between cholecystectomy/gallstones and the risk of developing malignant tumors. Our primary aim was to determine whether there is an increased likelihood of malignancy following gallstones/cholecystectomy, while also identifying specific types of tumors that may be causally associated with these conditions. This research is crucial for gaining deeper insights into the implications of cholecystectomy and for informing clinical decision-making.

## Methods and materials

### Study design and mendelian randomization assumptions

**Fig. 1:**
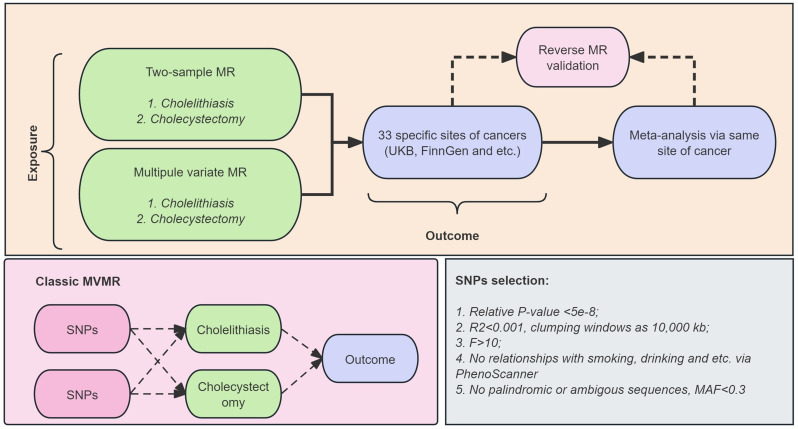
Diagram of work design and flow

Given the substantial overlap between populations undergoing cholecystectomy and those affected by cholelithiasis, and their reciprocal influence, this study aimed to investigate the direct impact of gallstones and cholecystectomy on the incidence of various cancers. Initially, the research employed MR, comparing a multivariable approach (MVMR) that combined gallstones and cholecystectomy as exposure factors, with univariable MR that assessed them individually. Subsequently, reverse MR was applied to mitigate reverse causation, ensuring more robust conclusions. The detailed flow diagram is presented in Fig. 1.

The effectiveness of the MR method relies on three key hypotheses: (1) instrumental variables are strongly associated with the exposure phenotypes and are mutually independent; (2) instrumental variables are not significantly associated with confounding factors that could influence both the exposure phenotypes and the outcome; and (3) instrumental variables cannot directly affect the outcome variables but only through their influence on the exposure phenotypes. The application of our multivariable MVMR and bidirectional MR is grounded in these fundamental principles.

### Data sources and instrumental variable selection

#### Data sources for cholelithiasis and cholecystectomy

The summary-level Genome-Wide Association Studies data for cholelithiasis and cholecystectomy were both sourced from the UK Biobank and subsequently made publicly available after processing by the Medical Research Council Integrative Epidemiology Unit, published on the “open GWAS” platform (https://gwas.mrcieu.ac.uk/). This dataset represents the largest collection of GWAS data related to gallstones and cholecystectomy among currently available European populations [34]. The dataset for gallstones included 7,682 cases and 455,251 controls, while the cholecystectomy dataset included 18,319 cases and 444,614 controls (detailed in the supplementary materials S6). The beta values are presented in standard deviations (SD) and can be converted into standardized log odds ratios using the method provided by the MRC-IEU, which involves division by u(1-u) (where u is the proportion of cases, calculated as ncase/(ncases + ncontrols)). The standard errors can also be transformed using the same methodology.

#### Data sources for 33 specific cancer sites

The FinnGen and UKB databases represent the most extensive and comprehensive publicly available GWAS databases to date. The FinnGen database encompasses summary information from over 400,000 Finnish individuals, covering more than 2,000 phenotypes, while the UKB includes data from approximately half a million participants from across the UK. The original data from the UKB are diverse (Pan-UKB team. https://pan.ukbb.broadinstitute.org2020), covering multiple ethnicities, a variety of biosamples, and detailed clinical and lifestyle information. We matched the summary-level cancer GWAS data from the FinnGen and UKB databases according to the type of cancer occurrence, supplemented missing cancer types with corresponding data from the GWAS Catalog and meticulously reviewed population information to prevent sample overlap issues (The final sample number in the analyses is explained in the supplementary materials S6).

This study included 33 cancer types as outcome indicators. For the respiratory system, malignancies of the larynx, bronchus, and lung were considered; the digestive tract included malignancies of the oral cavity, pharynx, esophagus, small intestine, stomach, pancreas, liver, extrahepatic bile ducts, gallbladder, and colorectal region; the hematopoietic and skeletal systems included multiple myeloma, Hodgkin’s lymphoma, non-Hodgkin lymphoma, acute myeloid leukemia, chronic myeloid leukemia, chronic lymphocytic leukemia, and bone-related malignancies; the reproductive system included malignancies of the ovary, cervix uteri, corpus uteri, vulva, breast, testis, and prostate; skin malignancies included malignant melanoma and nonmelanoma skin cancer; the nervous system included malignancies of the eye and its appendages and brain; the urinary system included malignancies of the kidney and bladder; and malignant neoplasms of the thyroid gland.

Furthermore, some tumor types did not utilize the original pan-UKB data but instead selected cases from other publicly available datasets with a larger number of cases. For instance, oral cavity cancer data (ncase = 1223, ncontrol = 2928) and oropharynx cancer data (ncase = 1090, ncontrol = 2928) were obtained from Lesseur C’s contribution to the GWAS Catalog with public data (GCST012237) [35]. Gallbladder and extrahepatic bile duct cancer (ncase = 195, ncontrol = 456,153), hepatocellular carcinoma (ncase = 123, ncontrol = 456,225) and intrahepatic cholangiocarcinoma (ncase = 104, ncontrol = 456,244) data were sourced from Jiang L’s contribution to the GWAS Catalog [18].

#### Instrumental variable selection

To adhere to the three crucial assumptions of Mendelian randomization, we implemented a stringent selection mechanism for SNPs as IVs. Initially, the SNPs were required to exhibit a strong correlation with the exposure factors, maintaining a p value threshold of less than 5e-8. To ensure the independence of the SNPs, linkage disequilibrium was meticulously assessed by calculating the R^2^ values based on the European population’s genetic variation map, with an R^2^ threshold of less than 0.001 and a physical distance greater than 10,000 kb to mitigate linkage disequilibrium. All palindromic or ambiguous SNPs were excluded. Additionally, to circumvent biases induced by weak instrumental variables, the F statistic was computed using the formula F = R^2 * [(*N* − 1 - k)/k] * (1 - R^2)[36], where only SNPs with an F value greater than 10 were considered for further analysis. Furthermore, the SNPs selected for analysis were scrutinized using the PhenoScanner database to exclude associations with carcinogenic factors such as smoking and alcohol consumption, thereby ensuring the robustness of the instrumental variables. Finally, we employed the MR-PRESSO package to conduct a leave-one-out analysis [37], eliminating significantly deviant SNPs to reduce heterogeneity.

### Mendelian randomization

In our univariable and reverse MR analyses, we employed the classical two-sample MR approach, utilizing methods such as inverse variance weighted [38], MR‒Egger, weighted median, simple mode, and weighted mode to assess causality. IVW has been validated as an efficacious method for causal inference in MR studies, providing a comprehensive estimate of all Wald estimates under the assumption of no significant pleiotropy, thus serving as the primary analytical approach in this research. In the absence of substantial pleiotropic evidence, the statistical significance of IVW is considered robust evidence of the final causal relationship. Furthermore, due to the high tolerance of MR‒Egger and weighted median methods for identifying invalid SNPs, they are also employed as crucial supplementary approaches to evaluate causality and are mainly used to assess the robustness of the MR causal direction and to contrast with IVW. The simple mode and weighted mode are included as supplementary analyses and are reported accordingly.

MVMR extends univariable MR, aiming to evaluate the direct effect relationship between an exposure and outcome after excluding significant confounders. In this study, all instrumental variable SNPs must show a strong correlation with at least one exposure, with other IV selection criteria as previously described, excluding SNPs associated with only one exposure. Additionally, an extended framework of IVW, MR‒Egger, and weighted median was used to conduct MVMR analyses on nonoverlapping samples for each exposure, yielding robust results.

The results postmeta-analysis are considered final, where positive findings are subjected to reverse MR analysis to avoid reverse bias, considering specific cancer types such as exposures and gallstones and cholecystectomy as outcomes. If the p value for IVW in the reverse MR was less than 0.05, reverse bias was present, and the results were deemed unreliable. The selection of IVs for reverse MR and the methods applied are consistent with those used in classical two-sample MR.

### Sensitivity and heterogeneity

In the sensitivity analysis, we primarily utilized the MR‒Egger and MR-PRESSO methods to evaluate potential horizontal pleiotropy. The MR‒Egger method robustly calculates the intercept of the group’s MR analysis and determines the potential p value of this intercept; a positive result implies the possibility of pleiotropy. Compared to the MR‒Egger method, the MR-PRESSO method more precisely calculates global pleiotropy and the pleiotropy of individual SNPs, thus excluding potential pleiotropic MR analyses. In this study, we considered the IVW results of the MR analysis to be reliable only if both the MR‒Egger and MR-PRESSO methods concurrently indicated the absence of pleiotropy.

For heterogeneity analysis, we computed the Cochrane Q value and its p value to assess the presence or absence of heterogeneity. We also constructed funnel plots for each group of MR analyses (detailed in the supplementary files), which subjectively determined the presence of heterogeneity. Moreover, in the MVMR analysis, we adopted a method to minimize the Q statistic, providing robust causal effect estimates even if the instruments are weak or exhibit pleiotropy, thereby enhancing the reliability of the results.

### Meta-analysis [39]

Due to the limited number of patients with some cancers, a postmeta-analytic approach was employed to enhance the robustness of our results. Specifically, after calculating the IVW results of MR for 33 types of cancer from the FinnGen database exposure data (both univariable and multivariable MR), these data were meta-analyzed with the IVW results from a validation set (primarily sourced from the UKB) for the same 33 cancer types, thereby obtaining more robust outcomes. Furthermore, MR results indicating horizontal pleiotropy were excluded from the meta-analysis. Positive results within the meta-analysis were subjected to further reverse MR analysis to eliminate the possibility of reverse bias.

### Statistical

All analyses were conducted on the R software platform (version 4.1.0), where MR analysis was primarily carried out using the “TwoSampleMR” package (version 0.5.6) and the “MendelianRandomization” package (version 0.9.0). Sensitivity analysis was performed with the “MR-PRESSO” package (version 1.0). Graph plotting and data processing were accomplished using packages such as “ggplot2” (version 3.4.4), “foreach” (version 1.5.2), and “data.table” (version 1.14.8). In MR analysis, when multiple testing was conducted, a p value less than 0.025 (0.05/2) after Bonferroni correction was considered to indicate a statistically significant causal association. Conversely, a p value between 0.025 and 0.05 indicated a potential causal relationship. Moreover, all analyses were two-tailed.

## Results

### Genetic instruments and strength

Having utilized the ieu-open-gwas tool to select instrumental variables for cholelithiasis and cholecystectomy phenotypes, we proceeded with a classical two-sample Mendelian randomization (MR) analysis. A robust set of 14 SNPs for cholelithiasis and 45 SNPs for cholecystectomy demonstrated strong F values (> 10), indicating minimal confounding bias or linkage disequilibrium. Detailed methodologies and the roles of these instrumental variables are provided in the supplementary materials.

### Discovery and replication results of cancer risk

Initiating with gallstones, we analyzed associations across multiple cancer types using data from FinnGen. In the analysis of gallstones and pan-cancer associations, the pan-cancer database sourced from FinnGen indicated that gallstones could increase the risk of hepatocellular carcinoma (P value: 0.005, OR = 1.26, 95% CI: 1.07–1.47), tumors of the lung and bronchus (P value: 0.009, OR = 1.07, 95% CI: 1.02–1.12), biliary system tumors (P value: 0.010, OR = 1.15, 95% CI: 1.03–1.27), hypopharyngeal tumors (P value: 0.018, OR = 1.58, 95% CI: 1.08–2.30), and nonmelanoma skin cancer (P value: 0.038, OR = 1.04, 95% CI: 1.00-1.07). The causal relationships between gallstones and these five specific site tumors maintained the same risk directionality across the IVW, weighted median, and MR‒Egger methods. In the validation pan-cancer dataset, which was primarily derived from the UKB dataset, significant differences were observed only for acute myelocytic leukemia (P value: 0.003, OR = 0.19, 95% CI: 0.07–0.56) and breast cancer (P value: 0.022, OR = 0.882, 95% CI: 0.79–0.98) due to the smaller sample sizes for many cancer types (such as liver cancer and biliary tract-related cancers); detailed results are available in the supplementary materials.

Similarly, analysis of individuals who underwent cholecystectomy, when combined with the pan-cancer database from FinnGen, revealed that cholecystectomy might lead to an increased risk of nonmelanoma skin cancer (P value: 0.038, OR = 1.03, 95% CI: 1.00-1.06), melanoma (P value: 0.008, OR = 1.07, 95% CI: 1.02–1.13), lung and bronchus tumors (P value: 0.028, OR = 1.06, 95% CI: 1.01–1.11), and bladder tumors (P value: 0.049, OR = 1.08, 95% CI: 1.00-1.16), with all three main analysis methods demonstrating consistency and robustness in the results’ directionality. However, in the validation set, the population that had undergone cholecystectomy did not show significant associations with tumors at specific sites (Table 1).

**Table 1:** Casual effect of gallstones and cholecystectomy on pan-cancer risk in a major cohort (FinnGen database) via univariate MR analysis

Outcome	Exposure	SNPs	Inverse variance weighted			MR-Egger intercept		Pleitropy	
			*p* value	OR	95% OR	Egger intercept	*p* value	MR-PRESSO.*p*-value	MR-Egeer.Q_pval
Biliary&gallbladder	cholecystectomy	42	0.541	1.038	0.921–1.170	-0.013	0.386	0.017	0.002
	cholelithiasis	14	0.010	1.147	1.034–1.274	-0.018	0.313	0.424	0.516
Bladder	cholecystectomy	42	0.049	1.075	1.000-1.156	0.008	0.386	0.763	0.721
	cholelithiasis	14	0.257	1.049	0.965–1.1409	0.010	0.464	0.597	0.417
Bone	cholecystectomy	42	0.626	1.056	0.849–1.314	0.014	0.600	0.543	0.464
	cholelithiasis	14	0.693	1.056	0.806–1.385	0.027	0.564	0.415	0.267
Brain	cholecystectomy	42	0.068	0.897	0.799–1.008	-0.006	0.679	0.445	0.364
	cholelithiasis	14	0.413	0.947	0.830–1.079	0.015	0.511	0.554	0.408
Breast	cholecystectomy	42	0.381	0.985	0.953–1.019	-0.004	0.279	0.022	0.009
	cholelithiasis	14	0.407	0.983	0.944–1.023	-0.004	0.515	0.123	0.034
Bronchus&lung	cholecystectomy	42	0.028	1.057	1.006–1.111	0.002	0.800	0.095	0.052
	cholelithiasis	14	0.009	1.068	1.017–1.122	0.006	0.453	0.752	0.803
Uterine cervix	cholecystectomy	42	0.451	1.070	0.897–1.276	0.002	0.936	0.310	0.232
	cholelithiasis	14	0.079	1.187	0.980–1.436	0.018	0.569	0.863	0.766
colorectal	cholecystectomy	42	0.221	1.043	0.975–1.114	0.005	0.519	< 0.001	0.000
	cholelithiasis	14	0.284	1.042	0.966–1.123	0.012	0.356	0.064	0.014
Uterine corpus	cholecystectomy	42	0.965	1.002	0.925–1.085	-0.004	0.699	0.242	0.163
	cholelithiasis	14	0.936	1.005	0.899–1.123	0.000	0.995	0.121	0.032
Eye&annexa	cholecystectomy	42	0.761	0.966	0.771–1.209	-0.018	0.515	0.433	0.357
	cholelithiasis	14	0.558	1.082	0.832–1.407	0.017	0.703	0.488	0.311
Liver cell carcinoma	cholecystectomy	42	0.067	1.150	0.990–1.334	0.001	0.959	0.194	0.188
	cholelithiasis	14	0.005	1.256	1.072–1.471	-0.015	0.597	0.949	0.961
Larynx	cholecystectomy	42	0.304	1.188	0.855–1.652	-0.073	0.068	0.331	0.561
	cholelithiasis	14	0.018	1.576	1.080–2.298	-0.036	0.567	0.832	0.826
Kidney	cholecystectomy	42	0.121	1.055	0.986–1.130	0.004	0.628	0.738	0.663
	cholelithiasis	14	0.559	1.024	0.945–1.111	-0.009	0.500	0.459	0.352
Malignant melanoma	cholecystectomy	42	0.008	1.072	1.018–1.128	0.004	0.496	0.839	0.760
	cholelithiasis	14	0.154	1.044	0.984–1.107	-0.008	0.438	0.656	0.755
Larynx	cholecystectomy	42	0.810	0.952	0.641–1.415	0.082	0.089	0.283	0.307
	cholelithiasis	14	0.840	0.957	0.622–1.471	0.070	0.337	0.763	0.824
Non-Hodgkin lymphoma	cholecystectomy	42	0.764	0.985	0.890–1.089	0.000	0.988	0.879	0.811
	cholelithiasis	14	0.458	0.957	0.852–1.075	-0.006	0.767	0.778	0.684
Esophagus	cholecystectomy	42	0.790	1.020	0.879–1.184	0.026	0.148	0.149	0.141
	cholelithiasis	14	0.826	1.021	0.849–1.227	0.027	0.382	0.244	0.124
Oralcavity	cholecystectomy	42	0.525	0.964	0.860–1.080	0.015	0.278	0.732	0.815
	cholelithiasis	14	0.227	0.922	0.808–1.052	0.014	0.513	0.699	0.746
Malignant nonmelanoma	cholecystectomy	42	0.038	1.032	1.002–1.064	-0.001	0.849	0.095	0.057
	cholelithiasis	14	0.038	1.036	1.002–1.071	0.001	0.855	0.440	0.198
Ovary	cholecystectomy	42	0.665	0.978	0.885–1.081	0.024	0.050	0.382	0.596
	cholelithiasis	14	0.176	0.922	0.820–1.037	0.018	0.362	0.493	0.389
Pancreas	cholecystectomy	42	0.840	1.009	0.921–1.106	0.010	0.367	0.176	0.121
	cholelithiasis	14	0.669	1.023	0.923–1.133	0.017	0.332	0.355	0.285
Prostate	cholecystectomy	42	0.377	1.022	0.974–1.071	0.002	0.758	< 0.001	0.000
	cholelithiasis	14	0.921	1.002	0.960–1.046	-0.003	0.635	0.270	0.110
Small intestine	cholecystectomy	42	0.327	1.075	0.930–1.242	0.013	0.476	0.487	0.413
	cholelithiasis	14	0.856	1.015	0.861–1.198	-0.018	0.516	0.599	0.482
Stomach	cholecystectomy	42	0.597	1.024	0.938–1.118	0.001	0.949	0.980	0.964
	cholelithiasis	14	0.533	1.033	0.933–1.143	0.003	0.874	0.846	0.723
Testis	cholecystectomy	42	0.810	0.981	0.835–1.151	-0.002	0.933	0.542	0.452
	cholelithiasis	14	0.833	0.979	0.805–1.190	-0.005	0.878	0.469	0.276
Thyroid	cholecystectomy	42	0.760	1.013	0.933-1.100	0.004	0.707	0.246	0.148
	cholelithiasis	14	0.125	0.933	0.855–1.019	-0.033	0.036	0.371	0.798
Vulva	cholecystectomy	42	0.626	1.059	0.841–1.333	-0.026	0.352	0.783	0.811
	cholelithiasis	14	0.739	1.046	0.802–1.364	-0.042	0.352	0.525	0.724
Hodgkin lymphoma	cholecystectomy	42	0.623	1.033	0.907–1.177	-0.008	0.629	0.085	0.061
	cholelithiasis	14	0.359	1.072	0.924–1.244	0.006	0.809	0.337	0.150
Multiple myeloma	cholecystectomy	42	0.924	1.004	0.917-1.100	-0.007	0.528	0.479	0.419
	cholelithiasis	14	0.481	1.043	0.928–1.172	0.002	0.928	0.373	0.172
Chronic lymphocytic leukemia	cholecystectomy	42	0.496	0.947	0.809–1.108	0.008	0.665	0.262	0.217
	cholelithiasis	14	0.731	0.968	0.803–1.166	0.026	0.412	0.313	0.252
Chronic myeloid leukemia	cholecystectomy	42	0.387	1.160	0.829–1.622	0.011	0.790	0.555	0.433
	cholelithiasis	14	0.795	1.053	0.715–1.550	-0.025	0.697	0.746	0.620
Oropharynx	cholecystectomy	42	0.297	1.106	0.915–1.337	0.059	0.008	0.173	0.392
	cholelithiasis	14	0.748	0.968	0.794–1.181	0.006	0.853	0.934	0.891
Acute myelocytic leukemia	cholecystectomy	42	0.265	1.159	0.895–1.501	-0.002	0.940	0.164	0.115
	cholelithiasis	14	0.067	1.350	0.979–1.860	0.095	0.062	0.234	0.314

All positive causal relationship results demonstrated no apparent pleiotropy in either MR‒Egger or MR-PRESSO sensitivity analyses, confirming the robustness and reliability of the IVW results.

### MVMR

Incorporating 35 SNPs, the MVMR analysis highlighted unique insights. Within the FinnGen-related pan-cancer dataset, after mutual adjustment of the cholelithiasis phenotype and the cholecystectomy phenotype, we observed that cholecystectomy played a role in increasing the risk for nonmelanoma skin cancer (P value < 0.001, OR = 1.63, 95% CI: 1.22–2.17), whereas cholelithiasis had the opposite effect (P value: 0.002, OR = 0.62, 95% CI: 0.46–0.84). Interestingly, in the MVMR analysis, small intestine tumors, which originally showed no significant association with either of the two exposure phenotypes in the previous two-sample MR, were significantly associated with both cholelithiasis and cholecystectomy in the MVMR, indicating an inverse relationship. Specifically, cholecystectomy was found to reduce the risk of small intestine tumors (P value: 0.014, OR = 0.16, 95% CI: 0.04–0.69), whereas the risk was increased in patients with gallstones (P value < 0.001, OR = 7.25, 95% CI: 1.61–32.61) (Table 2).

**Table 2:** Casual effect of gallstones and cholecystectomy on pan-cancer risk in a major cohort (FinnGen database) via multivariate MR analysis

Outcome	Exposure	SNPs	IVW-pvalue	OR	95% OR	IVW.Heterogeneity pvalue	MR-Egeer.Q_pval
Biliary&gallbladder	cholelithiasis	35.000	0.411	1.554	0.543–4.446	0.307	0.384
	cholecystectomy	35.000	0.540	0.730	0.267–1.995	0.307	0.384
Bladder	cholecystectomy	35.000	0.282	1.488	0.722–3.067	0.619	0.629
	cholelithiasis	35.000	0.353	0.699	0.328–1.488	0.619	0.629
Bone	cholecystectomy	35.000	0.142	5.179	0.577–46.487	0.600	0.580
	cholelithiasis	35.000	0.146	0.183	0.018–1.811	0.600	0.580
Brain	cholecystectomy	35.000	0.489	0.665	0.21–2.11	0.501	0.461
	cholelithiasis	35.000	0.595	1.387	0.415–4.636	0.501	0.461
Breast	cholelithiasis	35.000	0.352	0.856	0.616–1.188	0.059	0.057
	cholecystectomy	35.000	0.439	1.132	0.827–1.549	0.059	0.057
Bronchus&lung	cholecystectomy	35.000	0.077	1.606	0.949–2.716	0.033	0.027
	cholelithiasis	35.000	0.118	0.645	0.372–1.117	0.033	0.027
Uterine_cervix	cholelithiasis	35.000	0.468	1.921	0.329–11.209	0.447	0.399
	cholecystectomy	35.000	0.569	0.612	0.113–3.31	0.447	0.399
colorectal	cholecystectomy	35.000	0.352	1.401	0.689–2.848	0.000	0.000
	cholelithiasis	35.000	0.413	0.734	0.349–1.54	0.000	0.000
Uterine_corpus	cholecystectomy	35.000	0.706	1.181	0.497–2.805	0.078	0.064
	cholelithiasis	35.000	0.724	0.850	0.344–2.099	0.078	0.064
Eye&annexa	cholelithiasis	35.000	0.554	2.105	0.179–24.817	0.271	0.259
	cholecystectomy	35.000	0.557	0.493	0.047–5.226	0.271	0.259
Liver_cell_carcinoma	cholecystectomy	35.000	0.568	1.540	0.351–6.762	0.915	0.894
	cholelithiasis	35.000	0.781	0.803	0.171–3.775	0.915	0.894
Larynx	cholecystectomy	35.000	0.727	1.805	0.066–49.55	0.448	0.575
	cholelithiasis	35.000	0.834	0.691	0.022–22.057	0.448	0.575
Kidney	cholecystectomy	35.000	0.230	1.520	0.767–3.01	0.636	0.601
	cholelithiasis	35.000	0.297	0.684	0.335–1.397	0.636	0.601
Malignant_melanoma	cholecystectomy	35.000	0.148	1.460	0.874–2.439	0.796	0.784
	cholelithiasis	35.000	0.230	0.720	0.421–1.231	0.796	0.784
Larynx	cholelithiasis	35.000	0.734	2.017	0.035-114.636	0.402	0.482
	cholecystectomy	35.000	0.752	0.536	0.011–25.587	0.402	0.482
Non-Hodgkin_lymphoma	cholelithiasis	35.000	0.418	0.646	0.224–1.861	0.900	0.875
	cholecystectomy	35.000	0.444	1.485	0.54–4.085	0.900	0.875
Esophagus	cholelithiasis	35.000	0.682	1.408	0.273–7.257	0.067	0.086
	cholecystectomy	35.000	0.713	0.745	0.155–3.574	0.067	0.086
Oral_cavity	cholelithiasis	35.000	0.239	0.487	0.147–1.612	0.690	0.730
	cholecystectomy	35.000	0.277	1.887	0.6-5.931	0.690	0.730
Malignant_nonmelanoma	cholecystectomy	35.000	0.001	1.628	1.224–2.165	0.218	0.184
	cholelithiasis	35.000	0.002	0.621	0.461–0.836	0.218	0.184
Ovary	cholecystectomy	35.000	0.948	0.966	0.345–2.707	0.389	0.525
	cholelithiasis	35.000	0.978	1.015	0.346–2.98	0.389	0.525
Pancreas	cholecystectomy	35.000	0.370	1.531	0.604–3.881	0.130	0.131
	cholelithiasis	35.000	0.391	0.653	0.247–1.728	0.130	0.131
Prostate	cholecystectomy	35.000	0.153	1.429	0.876–2.33	0.000	0.000
	cholelithiasis	35.000	0.168	0.698	0.419–1.164	0.000	0.000
Small_intestine	cholelithiasis	35.000	0.010	7.250	1.612–32.607	0.611	0.577
	cholecystectomy	35.000	0.014	0.164	0.039–0.692	0.611	0.577
Stomach	cholecystectomy	35.000	0.881	1.069	0.443–2.58	0.955	0.941
	cholelithiasis	35.000	0.925	0.957	0.381–2.401	0.955	0.941
Testis	cholecystectomy	35.000	0.706	0.719	0.129–3.999	0.254	0.217
	cholelithiasis	35.000	0.734	1.365	0.227–8.201	0.254	0.217
Thyroid	cholelithiasis	35.000	0.879	0.933	0.381–2.284	0.131	0.111
	cholecystectomy	35.000	0.882	1.067	0.453–2.512	0.131	0.111
Vulva	cholecystectomy	35.000	0.416	2.623	0.256–26.861	0.820	0.818
	cholelithiasis	35.000	0.432	0.377	0.033–4.291	0.820	0.818
Hodgkin_lymphoma	cholelithiasis	35.000	0.953	1.041	0.271–3.997	0.122	0.104
	cholecystectomy	35.000	0.985	1.013	0.279–3.668	0.122	0.104
Multiple_myeloma	cholecystectomy	35.000	0.487	1.378	0.557–3.408	0.597	0.574
	cholelithiasis	35.000	0.509	0.727	0.282–1.873	0.597	0.574
Chronic_lymphocytic_leukemia	cholecystectomy	35.000	0.053	0.221	0.048–1.022	0.344	0.301
	cholelithiasis	35.000	0.059	4.685	0.944–23.263	0.344	0.301
Chronic_myeloid_leukemia	cholelithiasis	35.000	0.483	3.670	0.097-138.622	0.393	0.350
	cholecystectomy	35.000	0.524	0.323	0.01-10.427	0.393	0.350
Oropharynx	cholecystectomy	35.000	0.272	2.945	0.429–20.212	0.133	0.437
	cholelithiasis	35.000	0.325	0.364	0.049–2.722	0.133	0.437
Acute_myelocytic_leukemia	cholelithiasis	35.000	0.299	4.277	0.276–66.347	0.142	0.117
	cholecystectomy	35.000	0.343	0.281	0.02–3.869	0.142	0.117

However, in the pan-cancer analysis of the UKB, all results from the MVMR analysis were negative.

### Meta-analysis

A meta-analysis of FinnGen and UKB data confirmed increased risks associated with gallstones and cholecystectomy across various cancer types. After meta-analysis of the MR results for the pan-cancer cholecystectomy phenotype, the findings indicated that gallstones generally increased the risk of malignant nonmelanoma skin cancer (P-value: 0.047, OR = 1.03, 95%CI:1.00-1.06), melanoma (P-value: 0.005, OR = 1.07, 95%CI:1.02–1.13), and bladder tumors (P-value: 0.045, OR = 1.07, 95%CI:1.00-1.15). Conversely, the MR results for gallstones with pan-cancer phenotypes suggested that gallstones increase the risk of malignant nonmelanoma skin cancer (P value: 0.040, OR = 1.04, 95% CI: 1.00-1.07), lung tumors (P value: 0.009, OR = 1.07, 95% CI: 1.02–1.12), and biliary system tumors (P value: 0.003, OR = 1.15, 95% CI: 1.05–1.27). The aforementioned results suggest an increased risk of biliary system tumors due to cholecystectomy, a finding that seemingly contradicts common understanding and may indicate significant bias triggered by gallstones.

**Fig. 2:**
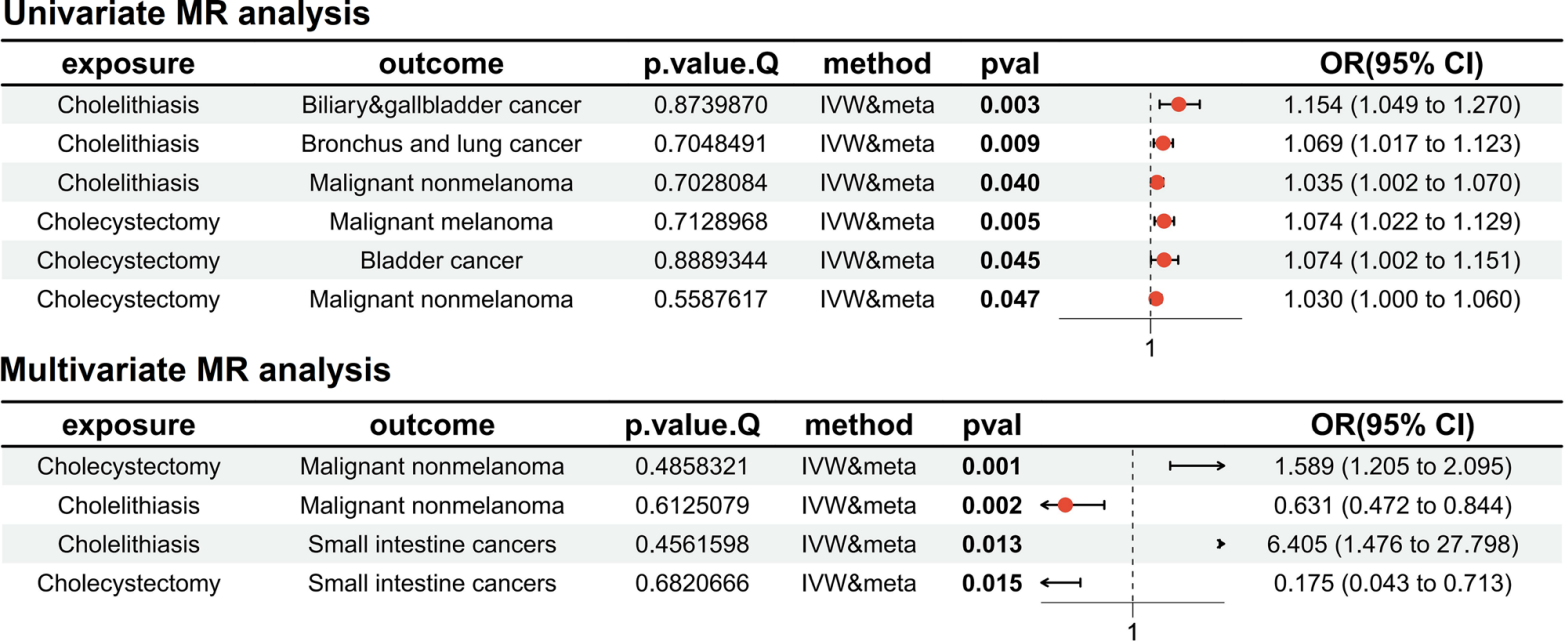
Forest plot of major positive results from meta-analysis of univariate Mendelian randomization (MR) and multivariable MR

In the MVMR analysis, after adjusting for gallstones, no significant differences were found, yet the results suggested that cholecystectomy could reduce the risk of biliary system tumors, whereas gallstones appeared to increase it. Moreover, the MVMR results were consistent with previous findings, indicating that cholecystectomy increases the risk of nonmelanoma skin cancer (P-value: 0.001, OR = 1.59, 95% CI: 1.20–2.10), whereas gallstones had the opposite effect (P-value: 0.002, OR = 0.63, 95% CI: 0.47–0.84); cholecystectomy was shown to reduce the risk of small intestine tumors (P-value: 0.015, OR = 0.18, 95% CI: 0.04–0.71), whereas gallstone patients had an increased risk (P-value: 0.013, OR = 6.41, 95% CI: 1.48–27.80). The details of the univariate and multivariate MR analyses are shown in Fig. 2, and more details can be found in the Supplementary files.

### Reverse MR

To address reverse causation, reverse MR analyses did not reveal significant reverse causal relationships, affirming the robustness of our meta-analysis findings (details in supplementary materials). For the positive results obtained after the meta-analysis, we employed a reverse MR approach to eliminate the possibility of reverse causation, with no apparent reverse causal relationships detected (based on the IVW method). Furthermore, all reverse MR analyses did not reveal any clear pleiotropy or heterogeneity. Based on these findings, we consider the meta-analysis results to be robust (detailed results are provided in the supplementary materials).

## Discussion

The present MVMR study systematically evaluated the causal associations between gallstones/cholecystectomy and 33 specific cancer sites throughout the body while accounting for potential interactions between gallstone disease and cholecystectomy. Our findings indicate that gallstones exert a positive effect on small intestinal tumors but confer a protective effect against nonmelanoma skin cancer. Furthermore, following cholecystectomy, patients experience a reduced risk of developing small intestinal tumors but an increased risk of developing nonmelanoma skin cancer.

Patients with gallstones demonstrated a 5.4-fold higher risk of developing small intestine cancer compared to those without gallstones (OR = 6.41, 95% CI: 1.48 to 27.80; *P* = 0.013). Interestingly, cholecystectomy significantly reduced the incidence of small intestinal tumors (OR = 0.18, 95% CI: 0.043 to 0.71; *P* = 0.015). Consistent with our findings, a previous large observational study in the U.S. population showed an increased risk of small-intestine carcinoid in gallstone patients, although they also reported an elevated risk post-cholecystectomy [24]. This could be attributed to the observational nature of the study and the lack of consideration for potential interactions between gallstones and cholecystectomy. Similarly, another extensive study from the United Kingdom found a significant association between gallstones and small intestine cancer, with the risk decreasing over time post-cholecystectomy, stabilizing after 8 to 10 years [40], aligning with our research. The primary function of the human gallbladder is to protect against hydrophobic bile acids (deoxycholic acid and lithocholic acid) [41, 42]. Gallstone formation increases the synthesis of these acids, thereby increasing the risk of intestinal tumors. Immediately following cholecystectomy, patients exhibit increased susceptibility to gastrointestinal tumors, particularly colorectal cancer [43]. Over time, as the gallbladder’s concentrating function diminishes and the common bile duct dilates, synthesis of hydrophobic bile acids decreases, consequently lowering the risk of intestinal tumors. This hypothesis warrants further validation through extensive prospective randomized controlled studies.

We also observed a protective effect of gallstones against nonmelanoma skin cancer occurrence (OR = 0.63, 95% CI: 0.47 to 0.84; *P* = 0.002). However, the risk of nonmelanoma skin cancer increased following cholecystectomy (OR = 1.59, 95% CI: 1.21 to 2.10; *P* = 0.001). This finding may be linked to metabolic changes post-cholecystectomy, potentially involving alterations in the bile acid pool and gut microbiome, which are known to influence systemic immune regulation and diarrhea [44–46]. Literature suggests that bile acid secretion affects the absorption of acitretin [47], a commonly used drug approved for nonmelanoma skin cancer prevention [48, 49]. Increased bile acid excretion post-cholecystectomy may reduce acitretin absorption, while diarrhea further impedes uptake of acitretin and its analogs, potentially contributing to the elevated risk of nonmelanoma skin cancer in post-cholecystectomy patients.

While the Mendelian randomization (MR) analysis of single exposure factors in our study did not establish definitive causality, it has revealed several intriguing findings that provide new insights into the relationship between gallstones, cholecystectomy, and extragallbladder cancers. In the single-exposure Mendelian randomization analysis, we identified a causal relationship between gallstones and bronchus/lung cancer (OR = 1.07, 95% CI: 1.02 to 1.12, *P* = 0.009), as well as with malignant nonmelanoma skin cancer (OR = 1.035, 95% CI: 1.002 to 1.070, *P* = 0.04). Limited research exists on the mechanisms linking gallstones to lung cancers. An autopsy-based study of 8,428 cases reported gallstones as a prevalent comorbidity among lung cancer patients, suggesting a potential correlation between these conditions [50]. This finding supports our investigation, proposing a plausible causal association between gallstones and lung cancer. However, case studies have also suggested that the occurrence of lung cancer in gallstone patients might result from surgical displacement of gallstones leading to thoracic inflammatory reactions and subsequent malignant transformation [51–53]. Furthermore, previous research indicates that changes in bile acid composition can activate FXR receptors in hepatic or intestinal epithelial cells, influencing inflammation and immune responses. These alterations may modify the tumor microenvironment, potentially promoting the development and progression of non-small cell lung cancer [54].

Additionally, our study revealed that cholecystectomy was linked to an increased risk of malignant melanoma (OR = 1.07, 95% CI: 1.02 to 1.13; *P* = 0.005). This finding is consistent with previous literature suggesting that the hydrophilic bile acid ursodeoxycholic acid, derived from animal bile, can induce apoptosis in human melanoma cells. Cholecystectomy may elevate levels of hydrophobic bile acids while reducing hydrophilic bile acids, potentially contributing to this increased risk [55].

Our study contributes significant insights into the intricate relationship between gallstones, cholecystectomy, and cancer risk across various organ sites. By systematically evaluating 33 specific cancer types, we have identified both positive and protective effects associated with gallstones and cholecystectomy, revealing potential mechanisms such as changes in bile acid composition and gut microbiome. These findings offer opportunities for targeted preventive strategies and personalized treatments in clinical practice. However, despite our comprehensive analysis, several knowledge gaps persist. Firstly, the precise mechanisms linking gallstones to specific cancer types, particularly involving bile acids and inflammatory responses post-cholecystectomy, require further elucidation. Additionally, understanding the impact of gallstone-related metabolic changes on cancer initiation and progression demands detailed investigation, possibly through prospective cohort studies with larger sample sizes and extended follow-up periods. Furthermore, unraveling the interplay among genetic predispositions, environmental factors, and gallstone disease could enhance understanding of cancer susceptibility and prognosis. Future research should prioritize: (i) Mechanistic studies to explore biological pathways linking gallstones, cholecystectomy, and cancer; (ii) Integration of genomic data with clinical outcomes to identify biomarkers for predicting cancer risk and early detection; (iii) Longitudinal studies to monitor cancer incidence among gallstone patients post-cholecystectomy, considering factors such as diet, lifestyle, and comorbidities.

Looking forward, the field of gallstone-related cancer research is poised for significant advancements. In the coming years, we anticipate: (i) Expanded use of Mendelian randomization and other causal inference methods to validate our findings and uncover new associations. (ii) Advances in genomic technologies that will enable more precise risk stratification and personalized therapeutic interventions. (iii) Development of targeted therapies leveraging insights into bile acid metabolism and microbiome changes. (iv) Collaborative efforts across disciplines to integrate epidemiological, molecular, and clinical data for comprehensive cancer risk assessment.

Our study has several limitations. First, while multiple sensitivity analyses were conducted, the potential influence of horizontal pleiotropy was not fully evaluated. Second, due to the lack of individual-level data, a comprehensive assessment of interactions such as those between gallbladder polyps and cholecystectomy could not be performed. Finally, despite employing cross-validation for robustness, this method may have missed potentially significant findings. Therefore, the conclusions drawn from this study should be interpreted cautiously, and further confirmation is necessary through prospective studies with larger sample sizes.

## Conclusion

In conclusion, our study highlights the intricate links between gallstones, cholecystectomy, and cancer risk, emphasizing the role of gallstones in elevating small intestinal tumor risk and the impact of cholecystectomy on nonmelanoma skin cancer patients. These findings advocate for cautious clinical management and underscore the urgent need for comprehensive prospective research to validate these associations and refine patient care protocols in the context of gallstone disease and post-cholecystectomy monitoring.

## Electronic supplementary material

Below is the link to the electronic supplementary material.

Supplementary Material 1

Supplementary Material 2

Supplementary Material 3

Supplementary Material 4

Supplementary Material 5

Supplementary Material 6

Supplementary Material 7

## Data Availability

Data is provided within the manuscript or supplementary information files.

## Abbreviations

GWAS: Genome-Wide Association Studies
MVMR: Multivariable Mendelian randomization
OR: Odds Ratio
CI: Confidence Interval
UKB: UK Biobank
FG: FinnGen
MR: Mendelian randomization
IVs: Instrumental Variables
SD: Standard Deviations
logORs: Log Odds Ratios
SEs: Standard Errors
MM: Multiple Myeloma
R: Correlation Coefficient
IVW: Inverse Variance Weighted
RCTs: Randomized Controlled Trials
SNPs: Single Nucleotide Polymorphisms
BMI: Body Mass Index
HDL: High-Density Lipoprotein
FXR: Farnesoid X Receptor
FGF15/19: Fibroblast Growth Factor 15/19
CYP7A1: Cholesterol 7 α-Hydroxylase
DCA: Deoxycholic Acid
LCA: Lithocholic Acid
MRC-IEU: Medical Research Council Integrative Epidemiology Unit
