# Supplementary material for: Investigating causal links between gallstones, cholecystectomy, and 33 site-specific cancers: a Mendelian randomization post-meta-analysis study
Source: BMC Cancer. 2024 Sep 27;24:1192. doi: 10.1186/s12885-024-12906-2 (PMC11437614; doi:10.1186/s12885-024-12906-2)
Supplement: Supplementary file 7 — Supplementary Material 7 [file 12885_2024_12906_MOESM7_ESM.doc]

| **Content** |
| --- |
| S1.1. The resluts of casual effect between cholecystectomy to the risk of pan-cancers in FinnGen database. |
| S1.2. The resluts of casual effect between cholecystectomy to the risk of pan-cancers in UKB database. |
| S1.3. The resluts of casual effect between cholelithiasis to the risk of pan-cancers in FinnGen database. |
| S1.4. The resluts of casual effect between cholelithiasis to the risk of pan-cancers in UKB database. |
| S2.1 The IVs SNPs involved in cholecystectomy to the risk of pan-cancers of FinnGen database. |
| S2.2 The IVs SNPs involved in cholecystectomy to the risk of pan-cancers of UKB database. |
| S2.3 The single SNP's effect estimated in cholecystectomy to the risk of pan-cancers of FinnGen database. |
| S2.4 The single SNP's effect estimated in cholecystectomy to the risk of pan-cancers of UK Biobank database. |
| S2.5 The IVs SNPs involved in cholelithiasis to the risk of pan-cancers of FinnGen database. |
| S2.6 The IVs SNPs involved in cholelithiasis to the risk of pan-cancers of UKB database. |
| S2.7 The single SNP's effect estimated in cholelithiasis to the risk of pan-cancers of FinnGen database. |
| S2.8 The single SNP's effect estimated in cholelithiasis to the risk of pan-cancers of UK Biobank database. |
| S3.1. The resluts of MVMR analysis between cholelithiasis&cholecystectomy to the pancancers in FinnGen database. |
| S3.2. The resluts of MVMR analysis between cholelithiasis&cholecystectomy to the pancancers in UKB database. |
| S4.1 The IVs SNPs involved in cholelithiasis&cholecystectomy to the risk of pan-cancers of FinnGen database. |
| S4.2 The IVs SNPs involved in cholelithiasis&cholecystectomy to the risk of pan-cancers of UKB database. |
| S4.3 The single SNP's effect estimated in cholelithiasis&cholecystectomy to the risk of pan-cancers of FinnGen database. |
| S4.4 The single SNP's effect estimated in cholelithiasis&cholecystectomy to the risk of pan-cancers of UK Biobank database. |
| S5.1 Meta analysis results of the casual effect of cholecystectomy and pan-cancers. |
| S5.2 Meta analysis results of the casual effect of cholelithiasis and pan-cancers. |
| S5.3 Meta analysis results of the casual effect of cholelithiasis&cholecystectomy and pan-cancers.  S6.1 The key GWAS data information of tumors from FinnGen databases.  S6.2 The key GWAS data information of tumors from other databases. |
